# Supplementary material for: A qualitative enquiry of health care workers’ narratives on knowledge and sources of information on principles of Respectful Maternity Care (RMC)
Source: PLOS Glob Public Health. 2023 Jan 6;3(1):e0000157. doi: 10.1371/journal.pgph.0000157 (PMC10021807; doi:10.1371/journal.pgph.0000157)
Supplement: Attachment — Submitted filename: REBUTTAL LETTER F.docx [file pgph.0000157.s002.docx]

RESPONSE TO REVIEWERS

PGPH-D-21-00476: A Qualitative Enquiry of Nurses Voices on Respectful Maternity Care (RMC) Principles.
PLOS Global Public Health


Along with the reviewers, I commend you for an article that takes on a critical topic. In revising your manuscript please pay careful attention to Reviewers #1 and #2's comments about the additional detail needed in the methods section. As the reviewers suggest, following the COREQ guidelines for reporting qualitative research and providing the interview agenda/coding scheme as appendices would significantly strengthen the paper and improve the likelihood of replication in other settings.

Please include the following items when submitting your revised manuscript:

- A rebuttal letter that responds to each point raised by the editor and reviewer(s). You should upload this letter as a separate file labeled 'Response to Reviewers'.
- A marked-up copy of your manuscript that highlights changes made to the original version. You should upload this as a separate file labeled 'Revised Manuscript with Track Changes'.
- An unmarked version of your revised paper without tracked changes. You should upload this as a separate file labeled 'Manuscript'.

**REBUTTAL LETTER**

Journal Requirements:

1. Please include a copy of the interview guide used in the study, in both the original language and English, as Supporting Information, or include a citation if it has been published previously.

- *We have cited our sister paper already published where we included the interview guide: Lusambili A, Wisofschi S, Shumba C, Obure J, Mulama K, Nyaga L, Wade TJ, Temmerman M. Health Care Workers' Perspectives of the Influences of Disrespectful Maternity Care in Rural Kenya. Int J Environ Res Public Health. 2020 Nov*
- *However, we have included the interview guide as an appendix to this copy but we do not plan to attach in the final publication because it is already published in the above journal.*

*Link: https://pubmed.ncbi.nlm.nih.gov/331721606;17(21):8218. doi: 10.3390/ijerph17218218. PMID: 33172160; PMCID: PMC7664382.*

- *All interviews were conducted English and we do not have any translations in another language as all the nurses’ we interviewed spoke English [Interview guide attached.*

 2. Please provide additional details regarding participant consent. In the ethics statement in the Methods and online submission information, please ensure that you have specified what type you obtained (for instance, written or verbal, and if verbal, how it was documented and witnessed).

 Thank you. We have addressed this in the main document to include “written consent”.

2. During your revisions, please note that a simple title correction is required: an apostrophe should be included after Nurses (i.e. ...of Nurses' Voices...). Please ensure this is updated in the manuscript file and the online submission information.

Thank you. This has been addressed. We have also corrected this throughout the manuscript

 4. We ask that a manuscript source file is provided at Revision. Please upload your manuscript file as a .doc, .docx, .rtf or .tex. If you are providing a .tex file, please upload it under the item type ‘LaTeX Source File’ and leave your .pdf version as the item type ‘Manuscript’.

5. We notice that your Appendix are included in the manuscript file.  Please remove them and upload them with the file type 'Supporting Information' . Please ensure that all Supporting Information files are included correctly and that each one has a legend listed in the manuscript after the references list.

We have addressed this.

 6. Since your data is not available for proprietary reasons, please explain via email why the data is not available. Please also include the contact information for the third party organization that should be contacted should other researchers want to request access to this data and please include the full citation of where the data can be found. We also request that you verify with us via email that any researcher will be able to obtain the data set in the same manner that the you have obtained it. If you feel you are unwilling or unable to adhere to this policy, please explain your reasons by return email and your exemption request will be escalated to the editor for approval. Your exemption request will be handled independently and will not hold up the peer review process, but will need to be resolved should your manuscript be accepted for publication. One of the Editorial team will be in touch if they require more information.

While we have the datasets for this study, we only interviewed 24 nurses across our two study areas who worked in the facilities that were part of AQCESS project interventions. These facilities were few and it is possible to link statements from this transcript to specific clinics and health care workers who were interviewees of this study. Also, in settings where we collected our data, many facilities are managed by two or three nurses and it is easy for other people to identify them. These concerns were raised by our participants before commencing the interviews and we assured them that information they provided will remain confidential. As access to the data could result in disclosure of participants, we are unable to provide access.

7. Please amend your] detailed Financial Disclosure statement. This is published with the article, therefore should be completed in full sentences and contain the exact wording you wish to be published.

This has been amended

i). State the initials, alongside each funding source, of each author to receive each grant.

This is previously stated under ‘MT’, senior author, who received the research grant.

ii). State what role the funders took in the study. If the funders had no role in your study, please state: “The funders had no role in study design, data collection and analysis, decision to publish, or preparation of the manuscript.”

The funders participated in the study design. This has been included.

iii) Please review your reference list to ensure that it is complete and correct. If you have cited papers that have been retracted, please include the rationale for doing so in the manuscript text, or remove these references and replace them with relevant current references. Any changes to the reference list should be mentioned in the rebuttal letter that accompanies your revised manuscript. If you need to cite a retracted article, indicate the article’s retracted status in the References list and also include a citation and full reference for the retraction notice.

Thanks this has been addressed.

Additional Editor Comments (if provided):

[Note: HTML markup is below. Please do not edit.]

Reviewers' comments:

Reviewer's Responses to Questions

**Comments to the Author**

1. Does this manuscript meet PLOS Global Public Health’s [publication criteria](https://eur03.safelinks.protection.outlook.com/?url=https%3A%2F%2Fjournals.plos.org%2Fglobalpublichealth%2Fs%2Fcriteria-for-publication&data=04%7C01%7Cadelaide.lusambili%40aku.edu%7Cedd1824355a2472a6c1c08d98e91a060%7Ca5d4252a02f94e6096f09733baae4919%7C0%7C0%7C637697578380665621%7CUnknown%7CTWFpbGZsb3d8eyJWIjoiMC4wLjAwMDAiLCJQIjoiV2luMzIiLCJBTiI6Ik1haWwiLCJXVCI6Mn0%3D%7C1000&sdata=d8%2FF%2FfZkXEV%2F3RbumJsTTW4AXuvmbckKLzBPffCnFS8%3D&reserved=0)? Is the manuscript technically sound, and do the data support the conclusions? The manuscript must describe methodologically and ethically rigorous research with conclusions that are appropriately drawn based on the data presented.

Reviewer #1: Yes

Reviewer #2: Partly

Reviewer #3: Yes

2. Has the statistical analysis been performed appropriately and rigorously?

Reviewer #1: Yes

Reviewer #2: N/A

Reviewer #3: N/A

3. Have the authors made all data underlying the findings in their manuscript fully available (please refer to the Data Availability Statement at the start of the manuscript PDF file)?

The [PLOS Data policy](https://eur03.safelinks.protection.outlook.com/?url=https%3A%2F%2Fjournals.plos.org%2Fglobalpublichealth%2Fs%2Fdata-availability&data=04%7C01%7Cadelaide.lusambili%40aku.edu%7Cedd1824355a2472a6c1c08d98e91a060%7Ca5d4252a02f94e6096f09733baae4919%7C0%7C0%7C637697578380665621%7CUnknown%7CTWFpbGZsb3d8eyJWIjoiMC4wLjAwMDAiLCJQIjoiV2luMzIiLCJBTiI6Ik1haWwiLCJXVCI6Mn0%3D%7C1000&sdata=FjkXAIwO3leSERjpL0ZqL8qARtHg50s1siKHHmlv%2BSI%3D&reserved=0) requires authors to make all data underlying the findings described in their manuscript fully available without restriction, with rare exception. The data should be provided as part of the manuscript or its supporting information, or deposited to a public repository. For example, in addition to summary statistics, the data points behind means, medians and variance measures should be available. If there are restrictions on publicly sharing data—e.g. participant privacy or use of data from a third party—those must be specified.

Reviewer #1: Yes

Reviewer #2: Yes

Reviewer #3: Yes

4. Is the manuscript presented in an intelligible fashion and written in standard English?

PLOS Global Public Health does not copyedit accepted manuscripts, so the language in submitted articles must be clear, correct, and unambiguous. Any typographical or grammatical errors should be corrected at revision, so please note any specific errors here.

Reviewer #1: Yes

Reviewer #2: Yes

Reviewer #3: Yes

5. Review Comments to the Author

Please use the space provided to explain your answers to the questions above. You may also include additional comments for the author, including concerns about dual publication, research ethics, or publication ethics. (Please upload your review as an attachment if it exceeds 20,000 characters)

Reviewer #1: PLOS Global PH Review: A Qualitative Enquiry of Nurses Voices on Respectful Maternity Care (RMC) Principles

An interesting study that provides important findings regarding respectful maternity care. Some comments to clarify and strengthen the manuscript below.
Backgrounds
Pg 2.
- Consistency re use of antenatal. In the manuscript, there is ‘ante-natal’, ‘ante natal’ and ‘antenatal’ – use what is used in the journal usually. Thanks. This has been addressed.
- Use HCWs in brackets first then use acronym. Thanks this has been addressed.
Methods:
- More information about the study setting in the study context – are these counties urban, rural, remote, population size, number of births a year. Thank you. In the study context we have cited our previous papers that have detailed information about this context. However, we have added some of this information in this manuscript without repeating what we have already published to avoid self-plagiarism.

- How many health facilities were staff recruited from, where these facilities large tertiary referral hospitals or smaller rural hospitals/health centres? This has been addressed.

- Note that the findings are reported according to the consolidated criteria for reporting qualitative research (COREQ) checklist – as you then provide as an appendix.

Data collection:
- Can you provide a copy of the interview guide as an appendix? I have attached an interview guide here for your review. However, we will not include interview guide with the manuscript as it is already published elsewhere. We have cited this in the paper (https://pubmed.ncbi.nlm.nih.gov/33172160/)

- Were HCWs quizzed on RMC components or did they offer this information voluntarily – what questions were they ask? – another reason to provide a copy of the interview guide. See the attached interview guides.

Data management:
- Did participants have an opportunity to review transcripts? No. A data interpretation workshop was held with some of the participants and key stakeholders across the two study areas.

Analysis:
- Can you provide a copy pf your coding framework in the appendix? Attached.

- Given many of your results are presented quantitatively, it sounds very much like a content analysis? Might be worth describing how this quantitative analysis took place

The percentages simply identify the proportion of participants who identified a specific RMC rights (Table 1) and sources for RMC information (Table 2) in relation to the total number of participants to make it easier to assess how well known each category is among HCWs. The results are not presented “quantitatively” as they are simple percentages. The reflect the extent to which each RMC right and source of RMC information is known among participants. We have adjusted the table column headings to reflect this.

Findings:
- Findings are reported against difference categories of quality care – would be useful to describe these in the background and in the methods discuss how these categories were applied to the findings of the qualitative analysis.

We have attached the interview guide and also described the questions in the methods.

- On page 6 – there is some more description about the rural regions with low literacy – would be good to embed this earlier on in the study setting/context.
- Also page 6, line 193 should be were poorly understand. Thanks this is addressed.

Discussion:
- In the discussion, it would be good to have greater unpacking of enablers and barriers to RMC

As we have noted this has already been addressed in our previous paper which we have cited in the section you are referring to. (<https://pubmed.ncbi.nlm.nih.gov/33172160/>) = *Lusambili A, Wisofschi S, Shumba C, Obure J, Mulama K, Nyaga L, Wade TJ, Temmerman M. Health Care Workers' Perspectives of the Influences of Disrespectful Maternity Care in Rural Kenya. Int J Environ Res Public Health. 2020 Nov 6;17(21):8218. doi: 10.3390/ijerph17218218. PMID: 33172160; PMCID: PMC7664382.*

- Study strength and limitations subheading should be bolded: Thanks. This is done

References:
- Consistency with respect to WHO or World Health Organization
- Review some of the references for grammar etc, inconsistent spacing between references
- Reference 13 is highlighted

Thank you. This is addressed.

Reviewer #2: The manuscript represents an important and timely topic of international interest, and is generally well presented. However, it has a number of weaknesses.

1. The authors reveal that this paper stems from a larger programme of research, in which there has been a series of papers. I do wonder whether the findings presented in this manuscript would have been better combined with a previous paper to provide greater evidence and critical discussion of the key points. By dissecting the work, these findings have less impact, as without referring back to the previous papers, it is difficult to see the whole picture.

As we have stated, the study had three objectives and each objective informed a paper. Please, see the study guide attached and the set of questions that were asked. Links to our previous papers are as follows.

1. <https://pubmed.ncbi.nlm.nih.gov/31910210/>
2. <https://pubmed.ncbi.nlm.nih.gov/33172160/>

2. The introduction could be strengthened by inclusion of recently published papers, with a clearer justification for the current study.
This has been done.

3. The manuscript does not follow COREQ reporting standards, thus there are many omissions. I would advise that this is followed. The methodology is limited; for example, there is no information on why a descriptive approach was adopted, why the sample size of 24 was chosen [was data saturation sought and gained?], what informed the interview guide? was the guide piloted?, and what was the recruitment strategy and process?

4. The study is based on the 2012 RMC Charter, but there is no rationale for using this particular charter as opposed to more recent ones, that have evolved over the last decade, e.g. WRA Charter 2018. This needs discussion. Thank you. This is well noted. The design of this study was an accumulation of a series of discussions since 2016 before the charter mentioned herewith. We have put this as a limitation and a reference for future research.

5. Where the interviewers known to the interviewees? Given that the interviews took place in the interviewees work place, and the subject area is very sensitive, this is particularly important. What, if anything, was done to mitigate against provision of desirable answers and/or any consequences of participation? Interviewees were not known to the facilitator and interviews were strictly held in private rooms.

6. Data analysis. This section does not include the use of NVIVO, whereas the abstract does. This needs correcting. This has been addressed.

7. Findings. In table 1 and 2, it is not usual to include percentages with small numbers.

As noted above, percentages have been provided to give a general idea with respect to the proportion of participants identifying a specific right or training source. When talking about, for example, 8 out of 24 participants, it is easier to indicate 1/3 of respondents which is all we are attempting to do here.

8. Findings. No characteristics have been provided for the included participants, therefore it is impossible to determine how transferable they are. A table would be needed to include participant factors such as years of professional training, professional group, prior RMC training, are of residence [Kilifi or Kisii] etc.

The main criteria for healthcare worker’s participations were: To have been working at the current facility for more than one year to provide firsthand accounts on the changes they had observed in the facilities following AQCESS project interventions. This has been included in the document. Due to the small number of participants, we are unable to provide individual-level characteristics to maintain confidentiality.

9. Findings. Although this is a descriptive qualitative study, one would still expect some further discussion around the quotes. In some places quotes are listed.

Thanks. This has been addressed.

10. The discussion could be strengthened by better integrating the findings into the current evidence base, and more explicitly highlighting what this paper brings that is new. The future research section lacks focus. Based on the findings, in combination with what we know from existing literature, we need research that not only increases our knowledge but translates that knowledge into action. This section needs revisiting.

**Being the first study to gauge HCWs knowledge on RMC in rural Kenya, kindly, could the reviewer provide suggestion on how this research (considering the small sample size) could help translate knowledge in to action without conducting further research?**

12. Limitations. The sites are atypical as they were programme implementation sites. This needs bringing out more in the limitations

The fact that these were implementation sites makes these findings all the more important because one would expect these sites to be more up-to-date on respectful and disrespectful care through participation with the program. The fact that these sites also have deficits in RMC knowledge identifies a larger, systemic issue that requires attention – as we try to do with this paper.

13. In places the language is not woman-friendly, such as use of the word 'patient' instead of 'woman', 'deliver' instead of 'birth' and 'antenatal and postnatal women', instead of 'women in the antenatal or postnatal period.' This has been addressed.

Reviewer #3: Thanks for the opportunity to review this manuscript on “Nurses Voices on Respectful Maternity Care (RMC) Principles.” This is an excellently written manuscript. After reading this manuscript, I have a few suggestions to make to the authors. Thank you.

The goal of the suggestion is to help them add an important aspect to their qualitative method.
It will be good when the authors add a section on how they dealt with the issue of trustworthiness (the role of the researchers and the issue of reflexivity) in their data collection and management. What’s the professional/academic background of the authors? Could their background have any influence on the way the data was generated and managed?

Thank you. This has been addressed.

Thank you.

6. PLOS authors have the option to publish the peer review history of their article ([what does this mean?](https://eur03.safelinks.protection.outlook.com/?url=https%3A%2F%2Fjournals.plos.org%2Fglobalpublichealth%2Fs%2Feditorial-and-peer-review-process%23loc-peer-review-history&data=04%7C01%7Cadelaide.lusambili%40aku.edu%7Cedd1824355a2472a6c1c08d98e91a060%7Ca5d4252a02f94e6096f09733baae4919%7C0%7C0%7C637697578380675582%7CUnknown%7CTWFpbGZsb3d8eyJWIjoiMC4wLjAwMDAiLCJQIjoiV2luMzIiLCJBTiI6Ik1haWwiLCJXVCI6Mn0%3D%7C1000&sdata=lw%2BQ2gSnYHIqhBOU%2BkwzYT957EMrNe7rzMEZiGLOgV4%3D&reserved=0)). If published, this will include your full peer review and any attached files.


**Do you want your identity to be public for this peer review?** If you choose “no”, your identity will remain anonymous but your review may still be made public.


For information about this choice, including consent withdrawal, please see our [Privacy Policy](https://eur03.safelinks.protection.outlook.com/?url=https%3A%2F%2Fwww.plos.org%2Fprivacy-policy&data=04%7C01%7Cadelaide.lusambili%40aku.edu%7Cedd1824355a2472a6c1c08d98e91a060%7Ca5d4252a02f94e6096f09733baae4919%7C0%7C0%7C637697578380675582%7CUnknown%7CTWFpbGZsb3d8eyJWIjoiMC4wLjAwMDAiLCJQIjoiV2luMzIiLCJBTiI6Ik1haWwiLCJXVCI6Mn0%3D%7C1000&sdata=BNQtsldSCh9Xbzvvqy90V%2FjODvQTAIFSJhjsw%2ByK5YQ%3D&reserved=0).

Reviewer #1: No

Reviewer #2: No

Reviewer #3: No

[NOTE: If reviewer comments were submitted as an attachment file, they will be attached to this email and accessible via the submission site. Please log into your account, locate the manuscript record, and check for the action link "View Attachments". If this link does not appear, there are no attachment files.]

While revising your submission, please upload your figure files to the Preflight Analysis and Conversion Engine (PACE) digital diagnostic tool, <https://pacev2.apexcovantage.com/>. PACE helps ensure that figures meet PLOS requirements. To use PACE, you must first register as a user. Registration is free. Then, login and navigate to the UPLOAD tab, where you will find detailed instructions on how to use the tool. If you encounter any issues or have any questions when using PACE, please email PLOS at [figures@plos.org](mailto:figures@plos.org). Please note that Supporting Information files do not need this step.

*In compliance with data protection regulations, you may request that we remove your personal registration details at any time.* [*(Remove my information/details)*](https://eur03.safelinks.protection.outlook.com/?url=https%3A%2F%2Fwww.editorialmanager.com%2Fpgph%2Flogin.asp%3Fa%3Dr&data=04%7C01%7Cadelaide.lusambili%40aku.edu%7Cedd1824355a2472a6c1c08d98e91a060%7Ca5d4252a02f94e6096f09733baae4919%7C0%7C0%7C637697578380685533%7CUnknown%7CTWFpbGZsb3d8eyJWIjoiMC4wLjAwMDAiLCJQIjoiV2luMzIiLCJBTiI6Ik1haWwiLCJXVCI6Mn0%3D%7C1000&sdata=fPNfv0twtKJcVl%2FXETL%2BOsCEsMWY%2FrQOQyaSfMhB3uk%3D&reserved=0)*. Please contact the publication office if you have any questions.*
