# Supplementary material for: A qualitative enquiry of health care workers’ narratives on knowledge and sources of information on principles of Respectful Maternity Care (RMC)
Source: PLOS Glob Public Health. 2023 Jan 6;3(1):e0000157. doi: 10.1371/journal.pgph.0000157 (PMC10021807; doi:10.1371/journal.pgph.0000157)
Supplement: Attachment — Submitted filename: Response to reviewers.docx [file pgph.0000157.s003.docx]

| **Date:** | Apr 11 2022 05:00PM |
| --- | --- |
| **To:** | "Adelaide M Lusambili" adelaide.lusambili@aku.edu |
| **cc:** | nicola.hawley@yale.edu |
| **From:** | "PLOS Global Public Health" globalpubhealth@plos.org |
| **Subject:** | PLOS Global Public Health Decision: Revision required [PGPH-D-21-00476R1] |

PGPH-D-21-00476R1
A qualitative Inquiry of Health Care Workers' Narratives on Knowledge and Sources of Respectful Maternity Care (RMC) Principles.
PLOS Global Public Health

Dear Dr. Lusambili,

Thank you for submitting your manuscript to PLOS Global Public Health. After careful consideration, we feel that it has merit but does not fully meet PLOS Global Public Health’s publication criteria as it currently stands. Therefore, we invite you to submit a revised version of the manuscript that addresses the points raised during the review process.

Thank you for your attention to the comments of the prior reviewers. They have just a few additional suggestions, which I agree will strengthen the submission. 

Please submit your revised manuscript by May 26 2022 11:59PM. If you will need more time than this to complete your revisions, please reply to this message or contact the journal office at globalpubhealth@plos.org. When you're ready to submit your revision, log on to https://www.editorialmanager.com/pgph/ and select the 'Submissions Needing Revision' folder to locate your manuscript file.

Please include the following items when submitting your revised manuscript:

- A rebuttal letter that responds to each point raised by the editor and reviewer(s). You should upload this letter as a separate file labeled 'Response to Reviewers'.
- A marked-up copy of your manuscript that highlights changes made to the original version. You should upload this as a separate file labeled 'Revised Manuscript with Track Changes'.
- An unmarked version of your revised paper without tracked changes. You should upload this as a separate file labeled 'Manuscript'.

Guidelines for resubmitting your figure files are available below the reviewer comments at the end of this letter.

We look forward to receiving your revised manuscript.

Kind regards,

Nicola L. Hawley
Academic Editor
PLOS Global Public Health

Journal Requirements:

**RESPONSE TO REVIEWERS**

1. Your co-authors:
   Stefania Wisfoschi -swisofschi@gmail.com
   Terrance J Wade -twade@brocku.ca
   Jerim Obure -Jobure@ijm.org
   ,have not confirmed authorship of the manuscript. We have resent them the authorship confirmation email; however please check that the above email address for them is correct and follow up personally to ensure they confirm.  Please note that we cannot proceed your manuscript until we have received confirmations from all co-authors.

**Thank you. All authors have confirmed authorship of the manuscript.**

Please review your reference list to ensure that it is complete and correct. If you have cited papers that have been retracted, please include the rationale for doing so in the manuscript text, or remove these references and replace them with relevant current references. Any changes to the reference list should be mentioned in the rebuttal letter that accompanies your revised manuscript. If you need to cite a retracted article, indicate the article’s retracted status in the References list and also include a citation and full reference for the retraction notice.

**All reference list has been reviewed. Thank you.**

Additional Editor Comments (if provided):


[Note: HTML markup is below. Please do not edit.]

Reviewers' comments:

Reviewer's Responses to Questions

**Comments to the Author**

1. If the authors have adequately addressed your comments raised in a previous round of review and you feel that this manuscript is now acceptable for publication, you may indicate that here to bypass the “Comments to the Author” section, enter your conflict of interest statement in the “Confidential to Editor” section, and submit your "Accept" recommendation.

Reviewer #2: (No Response)

**NOTED**

2. Does this manuscript meet PLOS Global Public Health’s [publication criteria](https://journals.plos.org/globalpublichealth/s/criteria-for-publication)? Is the manuscript technically sound, and do the data support the conclusions? The manuscript must describe methodologically and ethically rigorous research with conclusions that are appropriately drawn based on the data presented.

Reviewer #2: Yes

**Thank you.**

3. Has the statistical analysis been performed appropriately and rigorously?

Reviewer #2: N/A

**Noted**

4. Have the authors made all data underlying the findings in their manuscript fully available (please refer to the Data Availability Statement at the start of the manuscript PDF file)?

The [PLOS Data policy](https://journals.plos.org/globalpublichealth/s/data-availability) requires authors to make all data underlying the findings described in their manuscript fully available without restriction, with rare exception. The data should be provided as part of the manuscript or its supporting information, or deposited to a public repository. For example, in addition to summary statistics, the data points behind means, medians and variance measures should be available. If there are restrictions on publicly sharing data—e.g. participant privacy or use of data from a third party—those must be specified.

Reviewer #2: Yes

**Thank you.**

5. Is the manuscript presented in an intelligible fashion and written in standard English?

PLOS Global Public Health does not copyedit accepted manuscripts, so the language in submitted articles must be clear, correct, and unambiguous. Any typographical or grammatical errors should be corrected at revision, so please note any specific errors here.

Reviewer #2: Yes

**Thank you**

6. Review Comments to the Author

Please use the space provided to explain your answers to the questions above. You may also include additional comments for the author, including concerns about dual publication, research ethics, or publication ethics. (Please upload your review as an attachment if it exceeds 20,000 characters)

Reviewer #2: Thank you for the amendments made to this manuscript.

There are just a few outstanding considerations, which have either not been addressed/considered in the text or response to feedback.

Reviewer 2

**Point 3. It was requested that the authors provide rationale for:**

- Using a descriptive approach. This has been explained as follows “**Research on RMC is poorly understood in Kenya and employing a descriptive approach was deemed appropriate for this study since we sought to provide thick descriptions of HCWs narratives on this topic. Descriptive approach also provided flexibility in the analysis of the findings considering that our design was not theory driven”**
- The chosen sample size: Each ***HCWs participating in the study was selected in all facilities that AQCESS intervention took place. This has been included*.**
- Content of interview guide; this has not been done. **This has been addressed in lines 149-152 and we have also cited the study guide for reference.**
- The response to whether interviewees were known to facilitators needs to be included in the text, for the reader’s benefit. **We have added a sentence on this. Interviewers were not known to the interviewees.**

  Point 10. Apologies if this point was not clear. The authors state 'We recommend further qualitative research and quantitative research to probe all components of RMC as defined by the WHO to help better understand, identify and prioritise aspects of RMC that require intervention at the service delivery levels'. I was asking for this to be made a little stronger, given that the international community have expressed frustration on the continual exploration of RMC and publishing of rates, whilst interventions have been sparse. Consider whether your findings support future actions.

**I have added a sentence but perhaps we could benefit from your thoughts on how this could be strengthened. In addition, there isn’t much we can recommend from such a small qualitative research of knowledge and sources of information. More studies have reported on the existence of RMC and what is missing in the literature is whether HCWs have knowledge or understand the importance of RMC. This is what we have covered in this paper, and it could be a starting point to evoke discussions on possible interventions**.

Point 12. I agree with the response provided and suggest this goes in the manuscript.

**Yes, we have added this towards the end of the discussions**

7. PLOS authors have the option to publish the peer review history of their article ([what does this mean?](https://journals.plos.org/globalpublichealth/s/editorial-and-peer-review-process#loc-peer-review-history)). If published, this will include your full peer review and any attached files.


**Do you want your identity to be public for this peer review?** If you choose “no”, your identity will remain anonymous but your review may still be made public.


For information about this choice, including consent withdrawal, please see our [Privacy Policy](https://www.plos.org/privacy-policy).

Reviewer #2: No

Noted

[NOTE: If reviewer comments were submitted as an attachment file, they will be attached to this email and accessible via the submission site. Please log into your account, locate the manuscript record, and check for the action link "View Attachments". If this link does not appear, there are no attachment files.]

While revising your submission, please upload your figure files to the Preflight Analysis and Conversion Engine (PACE) digital diagnostic tool, https://pacev2.apexcovantage.com/. PACE helps ensure that figures meet PLOS requirements. To use PACE, you must first register as a user. Registration is free. Then, login and navigate to the UPLOAD tab, where you will find detailed instructions on how to use the tool. If you encounter any issues or have any questions when using PACE, please email PLOS at figures@plos.org. Please note that Supporting Information files do not need this step.

*In compliance with data protection regulations, you may request that we remove your personal registration details at any time.* [*(Remove my information/details)*](https://www.editorialmanager.com/pgph/login.asp?a=r)*. Please contact the publication office if you have any questions.*
