# Supplementary material for: A qualitative enquiry of health care workers’ narratives on knowledge and sources of information on principles of Respectful Maternity Care (RMC)
Source: PLOS Glob Public Health. 2023 Jan 6;3(1):e0000157. doi: 10.1371/journal.pgph.0000157 (PMC10021807; doi:10.1371/journal.pgph.0000157)
Supplement: Attachment — Submitted filename: Reviewers comments_TJW.docx [file pgph.0000157.s004.docx]

Title

"Sources of Respectful Maternity Care (RMC) Principles". This confusing; you could say "sources of information on respectful maternity care recommendations". There are no global principles but a charter which outlines the universal rights of childbearing women

**We have adjusted the title to alleviate any potential confusion. However, we did keep the word “principles” in the title as the analysis is based on the Charter. Thank you.**

Abstract

"respectful delivery of services" should be "respectful provision of services" and "labour" should be "childbirth". Please also use women-centred languages throughout; for example, use "gave birth" instead of "delivered". For more tips, please read <https://www.womenandbirth.org/article/S1871-5192(20)30088-3/fulltext>

**Thank you for identifying this. We have attempted to use more women-centred language throughout the manuscript.**

Instead of mentioning the software used for analysis, describe the method of analysis

**This has been addressed. Thank you.**

Introduction

I see the use of "respectful maternity care" and "disrespect and abuse" interchangeable. However, these two are different in terms of meaning and scope and must therefore be used logically. You could first start with the violation of women's rights during childbirth in health facilities (also termed as disrespect and abuse or mistreatment or obstetric violence) and them move to the strategy to mitigate disrespect and abuse - "respectful maternity care"

**Thank you for this comment. We have made revisions in the first paragraph to attempt to alleviate any confusion with the interchange of RMC and disrespectful maternal care.**

There are double spaces here and there.

**Thank you. This has been corrected throughout the manuscript.**

"In our previous papers, we provided an understanding of service users’ experiences and healthcare influences on these practices." What do you mean here? you mean the contribution of your study? if so, please revise the sentence. Additionally, cite the study

**We have attempted to rephrase this sentence to make it clearer. We have also cited our previous studies as appropriate in this section.**

"Yet there is limited research on what HCWs know and understand about RMC" Two points here 1) there is huge difference between knowledge and understanding; be specific to clearly describe what the aim of your study is 2) There is growing body of knowledge on service providers perceptions of respectful maternity care in SSA mainly Ethiopia, Ghana, Kenya, Nigeria, and Tanzania. I guess, your literature review was not thorough.

**Thank you. We have clarified the aim of the study to include both knowledge and understanding of RMC as well as where HCWs acquire this knowledge and training. With respect to the thoroughness of the literature review, we note in this paragraph that previous work (as you identify above) focuses on perceptions, not actual knowledge nor the sources of knowledge. This is the deficit in the literature that we are attempting to address with this paper. We hope we have made this clearer in the introduction.**

Methods

Move "Consolidated criteria for reporting qualitative studies (COREQ) checklist (attached) was used to report our findings" to the end of Data analysis sub-section if you are still interested to retain it.

**This has been done. Thank you.**

Move "As research on RMC is poorly understood in Kenya, we purposefully took this descriptive approach in an effort to provide thick descriptions of HCWs narratives on this topic." to the Introduction

**We have revised this sentence to refer specifically to methods. Thank you.**

Move the Ethics Approval to the end of the Methods section

**This has been done. Thank you.**

"Study sample participants and recruitment" is redundant and doesn't read well. You could say "sampling and recruitment"

**We have made this change.**

Interview protocol - first start with how the interview guides were developed, contents of the guide, and then go to how these were administered. Maintain the conventional logical order

**We have reorganized this section.**

In the Data analysis, please mention the method of analysis used. From what is written there, I think it is thematic analysis. If so, was it inductive or deductive or both? See also my comment in the Abstract

**Thank you. We have added this clarification to the data analysis section and abstract.**

"Categories were developed from the initial codes and merged into themes as illustrated in table 1 below." - you cannot cite the table here, but in the Results

**We have removed this from the methods section and added it to the beginning of the results.**

Results

Please add a descriptive section at the beginning highlighting profile of the key-informants (profession, experience, gender, etc )

**We have added this information to the beginning of the results (findings) section. Please note that much of this information is also contained in the methods section under the newly titled ‘sampling and recruitment’ section**

"The right of freedom from discrimination"should be "the right to......"

**This has been corrected. Thank you.**

Discussion

I found the Discussion This section should include critical reflections on the main findings of the study vis-a-vis the level of mistreatment during childbirth in health facilities in the study area, lessons from respectful maternity care packages implemented in similar settings (see countries mentioned in my earlier comment), other drivers of mistreatment and/or health system challenges in the promotion of respectful maternity care despite adequate knowledge among service provider, etc. Further in depth argument on the mismatch between knowledge and positive attitude is required to substantiate your point.

**We have highlighted some of these in our policy implementations and other drivers on of mistreatment are discussed in our reference 11 which we have acknowledged in this paper**

"antenatal and postnatal mothers" should be "pregnant and postpartum women"

**We have made these changes throughout the manuscript. Thank you.**

Have a second look at the addition in your Discussion (Line 499 - 503) "The fact that these findings are from the implementation the AQCESS project intervention sites makes these findings all the more important because one would expect that these sites to be more up-to-date on respectful and disrespectful care through participation with the program. If these sites also have deficits in RMC knowledge, it suggests a larger, systemic issue that are likely best addressed through national initiatives." "....all the more important".

**We have revised this section.**

Please make a thorough proofreading before we consider this paper for publication.

This has been done. Thanks you

[Note: HTML markup is below. Please do not edit.]

Reviewers' comments:

[NOTE: If reviewer comments were submitted as an attachment file, they will be attached to this email and accessible via the submission site. Please log into your account, locate the manuscript record, and check for the action link "View Attachments". If this link does not appear, there are no attachment files.]

While revising your submission, please upload your figure files to the Preflight Analysis and Conversion Engine (PACE) digital diagnostic tool, https://pacev2.apexcovantage.com/. PACE helps ensure that figures meet PLOS requirements. To use PACE, you must first register as a user. Registration is free. Then, login and navigate to the UPLOAD tab, where you will find detailed instructions on how to use the tool. If you encounter any issues or have any questions when using PACE, please email PLOS at figures@plos.org. Please note that Supporting Information files do not need this step.

In compliance with data protection regulations, you may request that we remove your personal registration details at any time. (Remove my information/details). Please contact the publication office if you have any questions.
