# Supplementary material for: A qualitative enquiry of health care workers’ narratives on knowledge and sources of information on principles of Respectful Maternity Care (RMC)
Source: PLOS Glob Public Health. 2023 Jan 6;3(1):e0000157. doi: 10.1371/journal.pgph.0000157 (PMC10021807; doi:10.1371/journal.pgph.0000157)
Supplement: Attachment — Submitted filename: Response to reviewers.docx [file pgph.0000157.s005.docx]

CC: [aasefa@itg.be](mailto:aasefa@itg.be)

PGPH-D-21-00476R3
A qualitative Inquiry of Health Care Workers' Narratives on Knowledge and Sources of Respectful Maternity Care (RMC) Principles.
PLOS Global Public Health

Dear Dr. Lusambili,

Thank you for submitting your manuscript to PLOS Global Public Health. After careful consideration, we feel that it has merit but does not fully meet PLOS Global Public Health’s publication criteria as it currently stands. Therefore, we invite you to submit a revised version of the manuscript that addresses the points raised during the review process.

Please submit your revised manuscript by Nov 04 2022 11:59PM. If you will need more time than this to complete your revisions, please reply to this message or contact the journal office at [globalpubhealth@plos.org](mailto:globalpubhealth@plos.org). When you're ready to submit your revision, log on to <https://www.editorialmanager.com/pgph/> and select the 'Submissions Needing Revision' folder to locate your manuscript file.

Please include the following items when submitting your revised manuscript:

- A rebuttal letter that responds to each point raised by the editor and reviewer(s). You should upload this letter as a separate file labeled 'Response to Reviewers'.
- A marked-up copy of your manuscript that highlights changes made to the original version. You should upload this as a separate file labeled 'Revised Manuscript with Track Changes'.
- An unmarked version of your revised paper without tracked changes. You should upload this as a separate file labeled 'Manuscript'.

Guidelines for resubmitting your figure files are available below the reviewer comments at the end of this letter.

We look forward to receiving your revised manuscript.

Kind regards,

Anteneh Asefa Mekonnen
Academic Editor
PLOS Global Public Health

Journal Requirements:

1. Please include a copy of the interview guide used in the study, in both the original language and English, as Supporting Information, or include a citation if it has been published previously.

- Authors have referenced the study guide on line 134 -135.
- Citation: Lusambili A, Wisofschi S, Shumba C, Obure J, Mulama K, Nyaga L, Wade TJ, Temmerman M. Health Care Workers' Perspectives of the Influences of Disrespectful Maternity Care in Rural Kenya. Int J Environ Res Public Health. 2020 Nov 6;17(21):8218. doi: 10.3390/ijerph17218218. PMID: 33172160; PMCID: PMC7664382.

2. Please provide additional details regarding participant consent. In the ethics statement in the Methods and online submission information, please ensure that you have specified what type you obtained (for instance, written or verbal, and if verbal, how it was documented and witnessed.

Thanks. This was reported in line 166 -169. The same information is already on the online submission.
Additional Editor Comments (if provided):

3. Thank you for the changes made in response to my comments. The manuscript reads better now. However, The Discussion is still shallow and lacks rigour to substantiate the findings of your study in light with current evidence in the field of respectful maternity care specifically and quality of maternal care generally, of course focusing on providers perspective. I was surprised to see again you have just 13 references, only 3 used in the Discussion. If you are experiencing difficulty identifying relevant resources/articles which could be used to strengthen the Discussion, I can provide you with many. In the first paragraph, be specific - not "care" but "respectful maternity" care.

We have added some references. Important to note, there isn’t much research on HCWs sources and information on principles of RMC. However, there is much literature on the influences, nature and type and experiences of respectful maternity care, as you have rightfully indicated.
4. The Methods sub-section of the Abstract should indicate the method of data analysis and frameworks used (if any)

Both deductive and inductive analysis was used. Braun and Clark framework was applied. This was already reported in line 194-200


[Note: HTML markup is below. Please do not edit.]

Reviewers' comments:


[NOTE: If reviewer comments were submitted as an attachment file, they will be attached to this email and accessible via the submission site. Please log into your account, locate the manuscript record, and check for the action link "View Attachments". If this link does not appear, there are no attachment files.]

While revising your submission, please upload your figure files to the Preflight Analysis and Conversion Engine (PACE) digital diagnostic tool, <https://pacev2.apexcovantage.com/>. PACE helps ensure that figures meet PLOS requirements. To use PACE, you must first register as a user. Registration is free. Then, login and navigate to the UPLOAD tab, where you will find detailed instructions on how to use the tool. If you encounter any issues or have any questions when using PACE, please email PLOS at [figures@plos.org](mailto:figures@plos.org). Please note that Supporting Information files do not need this step.

*In compliance with data protection regulations, you may request that we remove your personal registration details at any time.* [*(Remove my information/details)*](https://eur03.safelinks.protection.outlook.com/?url=https%3A%2F%2Fwww.editorialmanager.com%2Fpgph%2Flogin.asp%3Fa%3Dr&data=05%7C01%7Cadelaide.lusambili%40aku.edu%7C9c6b62a5028548d2f26308da9b5e5e03%7Ca5d4252a02f94e6096f09733baae4919%7C0%7C0%7C637993126847643707%7CUnknown%7CTWFpbGZsb3d8eyJWIjoiMC4wLjAwMDAiLCJQIjoiV2luMzIiLCJBTiI6Ik1haWwiLCJXVCI6Mn0%3D%7C3000%7C%7C%7C&sdata=0DuWkcwei9Ezr6hb2oBwzgNF1y33yhe9QxKinvr5RCA%3D&reserved=0)*. Please contact the p*
